# Extracellular Vesicles, Liposomes, and Hybrid Nanovesicles: Comparative Strategies for Targeted Cancer Therapy

**DOI:** 10.3390/ijms27135795

**Published:** 2026-06-26

**Authors:** Alessia Brossa, Michela Arena, Elena Ceccotti, Enza Di Gregorio, Giuseppe Ferrauto, Benedetta Bussolati, Stefania Bruno

**Affiliations:** 1 Department of Molecular Biotechnology and Health Science, University of Torino, 10126 Torino, Italy; alessia.brossa@unito.it (A.B.); or michela.arena@unina.it (M.A.); enza.digregorio@unito.it (E.D.G.); giuseppe.ferrauto@unito.it (G.F.); 2 Department of Pharmacy, University of Naples Federico II, 80131 Naples, Italy; 3 Department of Medical Sciences, University of Torino, 10126 Torino, Italy; elena.ceccotti@unito.it (E.C.); benedetta.bussolati@unito.it (B.B.)

**Keywords:** drug delivery systems, tumor targeting, cancer nanomedicine

## Abstract

Extracellular vesicles (EVs) and liposomes are nanoscale drug delivery systems extensively investigated in oncology for their ability to improve pharmacokinetics, biodistribution, and therapeutic efficacy of anticancer agents. Liposomes are clinically validated synthetic nanocarriers characterized by high versatility, scalable production, and established regulatory approval; however, their performance is limited by tumor heterogeneity, vascular barriers, adverse effects and inefficient intracellular drug release. EVs are naturally derived nanoparticles involved in intercellular communication and exhibit intrinsic biocompatibility, low immunogenicity, and biological targeting potential; yet their translation is constrained by heterogeneity, limited loading capacity, and manufacturing challenges. Different studies indicate complementary advantages between both systems, with EVs favoring biological targeting and immune modulation and liposomes enabling controlled formulation and pharmacokinetic optimization. These features have driven the development of hybrid EV–liposome nanovesicles, which integrate synthetic and biological properties to enhance tumor targeting, therapeutic efficacy, and payload diversity, including drugs, nucleic acids, and gene-editing systems. Despite promising preclinical results, challenges remain in scalability, standardization, and mechanistic understanding of in vivo behaviour. Overall, these hybrid strategies represent a promising platform for next-generation precision nanomedicine in cancer therapy and for advancing clinical translation by addressing key limitations of current delivery systems and improving therapeutic index and patient outcomes.

## 1. Introduction

Liposomes and extracellular vesicles (EVs) are nanocarriers that allow for the targeted delivery of therapeutic agents due to their ability to encapsulate a wide range of active substances, improving the therapeutic efficacy while minimizing systemic toxicity in different tumors [1,2].

Liposomes represent one of the most extensively investigated and clinically validated classes of biocompatible nanosystems for drug delivery, with established applications in oncology. Since their first description in the 1960s, they have attracted sustained scientific and clinical interest due to their compositional flexibility and ability to modulate the pharmacokinetics of therapeutic agents. Their continued relevance in nanomedicine is reflected by the number of liposomal formulations that have progressed from preclinical development to clinical approval [3,4,5].

While liposomes remain a clinically validated benchmark in nanomedicine, their limited capacity to overcome certain biological barriers highlights the need for alternative and complementary delivery approaches. EVs, as naturally occurring nanoscale messengers, have recently emerged as a promising alternative drug delivery system and may address some of these limitations. Considering their natural origin, EVs exhibit intrinsic biocompatibility, low immunogenicity, and the ability to participate in intercellular communication both in physiological and pathological conditions [1,6]. Compared with synthetic nanoparticles, EVs exhibit high biocompatibility, reduced immunogenicity, and an inherent ability to interact with specific recipient cells through membrane-associated ligands. Moreover, EV membranes provide partial protection of encapsulated cargo from enzymatic degradation in biological fluids. However, the translational development of EV-based delivery systems remains challenged by several factors, including heterogeneous vesicle populations, limited drug loading capacity, and difficulties in large-scale production [7]. The convergence of synthetic and biological delivery strategies has therefore prompted the development of hybrid liposome–EV systems aimed at combining structural precision with intrinsic biological functionality.

Although several reviews have separately discussed liposomes [4,5], EVs [7], or hybrids [8,9,10], most have focused predominantly on formulation strategies, EV biology, or specific therapeutic applications. The present review provides an integrated analysis of liposomes, EVs, and hybrid EV–liposomes within a unified oncological framework. Particular emphasis is placed on (i) the mechanistic comparison of cellular uptake and intracellular trafficking pathways, (ii) the translational limitations restricting clinical efficacy of both systems, and (iii) the emerging rationale behind hybrids as a strategy to combine synthetic controllability with biological targeting functions. Furthermore, this review highlights the limited number of direct head-to-head studies comparing EVs and liposomes in oncology, discussing how these comparative data support the development of next-generation hybrid delivery platforms.

### Literature Search Strategy

A systematic literature search was conducted to identify all relevant published studies comparing EVs and liposomes in different oncological in vitro and in vivo models, as well as studies supporting the development of next-generation hybrid delivery platforms. The electronic database PubMed was searched from its inception until March 2026. Only studies published in English were included. The following keywords were used: “liposomes and extracellular vesicles”, “comparison”, and “in vitro and in vivo tumor models”. Studies investigating only liposomes or extracellular vesicles without a direct comparative evaluation were excluded. The screening process involved an initial review of titles and abstracts, followed by a full-text assessment of potentially eligible articles.

## 2. Liposome Structure and Functionalization

**Figure 1:**
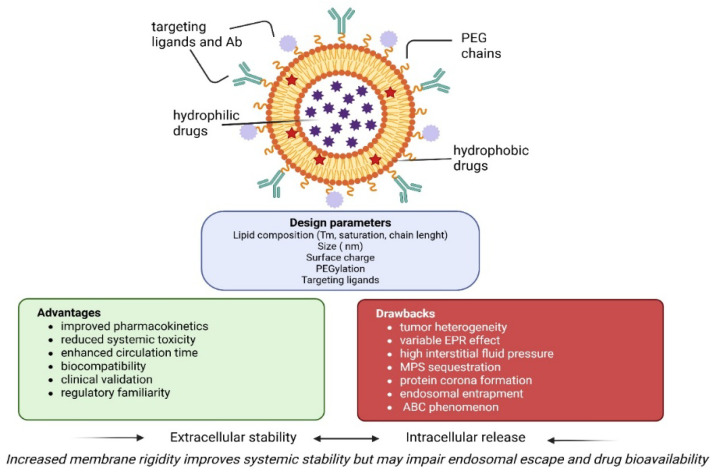
Structural features, clinical advantages, and biological constraints of liposomal drug delivery systems in oncology. Liposomes offer design flexibility and improved pharmacokinetics but face intrinsic biological barriers within the tumor microenvironment, including heterogeneous vascular permeability, MPS sequestration, and limited intracellular drug release. The balance between systemic stability and intracellular bioavailability represents a key design trade-off. EPR: Enhanced Permeability and Retention, MPS: mononuclear phagocyte system, ABC: accelerated blood clearance.

From a chemical and structural perspective, liposomes consist of one or more concentric phospholipid bilayers enclosing an aqueous core [5,11] (Figure 1). Based on size and lamellarity, liposomes are classified as small unilamellar (SUVs, <100 nm), large unilamellar (LUVs, 100–1000 nm), or multilamellar vesicles (MLVs, >500 nm). The amphiphilic nature of phospholipids allows liposomes to encapsulate hydrophilic drugs within the aqueous core and hydrophobic or amphipathic molecules within the lipid bilayer, providing a versatile dual-loading capacity. Lipid composition can be precisely tuned using natural or synthetic phospholipids differing in headgroup polarity, acyl chain length, and degree of saturation, often combined with cholesterol to regulate membrane rigidity, permeability, and stability. Lipid phase behavior, including transition temperature (Tm) and bilayer fluidity, critically influences drug retention and release kinetics. High-Tm saturated lipids (e.g., DPPC or DSPC) and cholesterol-rich formulations enhance drug retention but may limit membrane fusion and intracellular release. Overall, liposome size, surface charge, and membrane fluidity strongly determine circulation time, tumor penetration, and cellular uptake [5]. A major strength of liposomal systems lies in their adaptable surface functionalization (Figure 1). PEGylation, one of the most widely used strategies, reduces opsonization and uptake by the mononuclear phagocyte system (MPS), thereby prolonging circulation and promoting passive tumor accumulation. Beyond stealth properties, liposomes can be actively targeted through surface conjugation of antibodies, peptides, aptamers, or small molecules to enhance receptor-mediated uptake (Figure 1). Advances in bioconjugation, including orthogonal and click chemistry approaches, enable controlled and site-specific ligand attachment, allowing precise control over ligand density and orientation while preserving liposome integrity and translational reproducibility.

### Applications of Liposomes in Oncology

In oncological applications, liposome particle size typically ranges between 80 and 200 nm, optimizing biodistribution and tumor accumulation via the enhanced permeability and retention (EPR) effect. While smaller vesicles may display prolonged circulation and improved extravasation, they can exhibit reduced loading capacity for hydrophilic drugs [11,12,13].

Liposomes have achieved their greatest impact in oncology by improving the pharmacokinetic profile and safety of cytotoxic agents rather than by fundamentally altering their antitumor mechanisms of action. In particular, liposomes can (i) prolong drug circulation time, (ii) improve the stability of therapeutic agents in biological fluids, (iii) increase drug accumulation at target sites through passive or active targeting, and (iv) reduce off-target toxicity in healthy tissues, such as the heart [14] (Figure 1).

Several liposomal formulations have successfully reached clinical practice, including PEGylated liposomal doxorubicin (Doxil^®^/Caelyx^®^), liposomal daunorubicin (DaunoXome^®^), liposomal irinotecan (Onivyde^®^), and the fixed liposomal combination of daunorubicin and cytarabine (Vyxeos^®^). These approved products clearly demonstrate the capacity of liposomal encapsulation to improve the pharmacokinetic profile and tolerability of chemotherapeutic agents. However, their effect on overall survival has generally been indication-dependent and, in many solid tumors, limited in magnitude. As a result, liposomes have established themselves as a robust and clinically validated drug delivery platform whose therapeutic impact remains constrained by biological, pharmacokinetic, and translational barriers associated with the complexity and heterogeneity of human tumours. On the other side, effective in reducing the intrinsic toxicity of encapsulated drugs, liposomes may paradoxically trigger new adverse effects including infusion-related reactions (chills, fever, dizziness), hand-foot syndrome, and immunological reactions such as complement system activation [15,16,17].

Tumour vascularization is highly variable not only among different cancer types and patients, but also within distinct regions of the same tumour, severely limiting the predictability and uniformity of liposomal accumulation even for formulations optimized for passive targeting. The enhanced EPR effect, which underpins most liposomal oncology strategies, is a particularly critical bottleneck (Figure 1). Although leaky vasculature and impaired lymphatic drainage are commonly observed in murine tumor models, the magnitude and consistency of the EPR effect in human cancers are far less pronounced and highly patient-dependent. As a result, liposomal formulations that demonstrate robust tumor accumulation and antitumor efficacy in preclinical models often fail to reproduce these outcomes in clinical settings [18].

In addition, elevated interstitial fluid pressure, a hallmark of many solid tumors, further hampers the extravasation and deep penetration of liposomes into the tumor parenchyma. Consequently, liposomes tend to accumulate predominantly in perivascular regions, leading to heterogeneous intratumoral distribution and limited access to hypoxic or poorly vascularized tumor areas. Dense extracellular matrix components, cancer-associated fibroblasts, and infiltrating immune cells constitute additional physical and biological barriers that restrict liposomal transport and cellular access [19].

Another fundamental limitation of liposomal systems concerns drug release and bioavailability. Tumor accumulation of the carrier does not necessarily translate into effective drug delivery at the cellular level. Many liposomal formulations release their payload either too slowly at the tumor site or prematurely in systemic circulation, resulting in suboptimal drug exposure of cancer cells [20]. Following systemic administration, a substantial fraction of liposomes is sequestered by the MPS, particularly in the liver and spleen (Figure 1). Although PEGylation can reduce opsonization and prolong circulation time, MPS uptake remains a major clearance pathway for liposomal formulations. Moreover, the development of an immune response and immunoglobulin M (IgM) formation may promote the rapid clearance of repeated liposome doses from the bloodstream, resulting in diminished therapeutic efficacy, the so-called accelerated blood clearance (ABC) phenomenon [21].

## 3. Biology of Extracellular Vesicles

EVs are nanoscale membrane-bound particles naturally released by virtually all cell types and involved in intercellular communication under both physiological and pathological conditions. EVs transport a diverse molecular cargo, including proteins, lipids, DNA fragments, messenger RNAs, and regulatory non-coding RNAs, thereby mediating the transfer of functional biomolecules between donor and recipient cells [1,22]. Through this mechanism, EVs contribute to the regulation of immune responses, tissue homeostasis, and disease progression, including tumor growth and metastasis.

EVs are commonly classified into three main subtypes based on their size and biogenesis pathways: exosomes (30–150 nm), microvesicles (100–1000 nm), and apoptotic bodies (>1000 nm). Exosomes originate from the endosomal system and are generated through the inward budding of the limiting membrane of multivesicular bodies (MVBs), which subsequently fuse with the plasma membrane and release intraluminal vesicles into the extracellular space. In contrast, microvesicles are produced through direct outward budding of the plasma membrane, a process driven by cytoskeletal rearrangements and changes in membrane lipid asymmetry. Apoptotic bodies arise during programmed cell death and contain cellular organelles and fragmented genomic material. The formation of intraluminal vesicles within MVBs is primarily regulated by the endosomal sorting complex required for transport (ESCRT) machinery, although ESCRT-independent mechanisms involving lipid microdomains, ceramide generation, and tetraspanin-enriched membrane regions have also been described. These molecular sorting pathways contribute to the selective loading of proteins, nucleic acids, and lipids into EVs, resulting in vesicles that partially reflect the molecular composition and physiological state of the parent cell. EV membranes are enriched in cholesterol, sphingolipids, and phosphatidylserine, which contribute to membrane stability and influence interactions with recipient cells. In addition, EVs display characteristic membrane proteins such as tetraspanins (CD9, CD63, CD81), integrins, heat shock proteins, and major histocompatibility complex molecules. These surface components play an important role in determining EV biodistribution, cellular recognition, and uptake mechanisms [22,23,24].

Within the tumor microenvironment, EVs contribute to multiple aspects of cancer progression. Tumor-derived EVs can modulate the surrounding stroma, promote angiogenesis, suppress immune responses, and facilitate the formation of pre-metastatic niches in distant organs. At the same time, EVs released by immune cells, stromal cells, and endothelial cells participate in complex bidirectional signalling networks that shape tumor evolution [25].

## 4. Uptake and Intracellular Trafficking of EVs and Liposomes

EVs and liposomes share structural similarities but differ in biological origin, molecular composition, and interactions with recipient cells [26,27]. These differences influence cellular uptake mechanisms and intracellular trafficking pathways, which ultimately affect therapeutic efficacy [28,29]. Cellular internalization generally occurs through energy-dependent endocytic mechanisms, although multiple uptake routes can coexist within the same cell [30,31].

**Figure 2:**
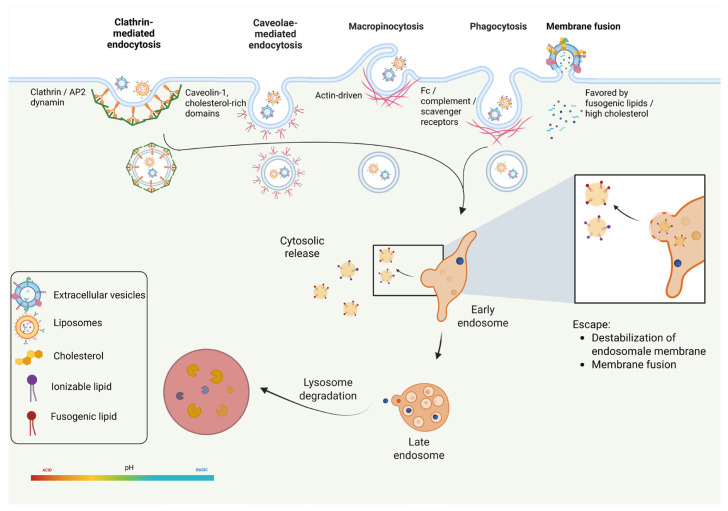
Schematic representation of uptake and trafficking of EVs and liposomes: EVs and liposomes can enter recipient cells through multiple uptake pathways, including endocytosis and, less frequently, direct membrane fusion. Following internalization, vesicles traffic through the endo-lysosomal pathway (early and late endosomes), where cargo may undergo lysosomal degradation unless endosomal escape occurs. Escape into the cytosol can be mediated by membrane destabilization or fusion processes, enabling the release of intracellular cargo. Differences in membrane composition and physicochemical properties influence uptake routes, intracellular trafficking, and delivery efficiency.

In recent years, increasing attention has been devoted to the role of the protein corona in regulating cellular uptake. Upon exposure to serum or plasma, lipid nanocarriers rapidly acquire a dynamic protein corona that alters their biological identity and modulates receptor engagement at the cell surface, thereby influencing uptake routes and intracellular trafficking [32,33,34]. These observations collectively suggest that the selection of uptake routes is not inherently determined by EVs or liposomes themselves. Instead, it arises from the interaction between the physicochemical properties of the vesicles, their surface composition, and features of the recipient cell (Figure 2).

### 4.1. Clathrin-Mediated Endocytosis

Clathrin-mediated endocytosis (CME) is one of the best-characterized internalization pathways [35]. In this process, clathrin and adaptor proteins assemble at the plasma membrane to form coated pits that invaginate and undergo dynamin-dependent scission, generating ~100 nm vesicles that subsequently traffic to early endosomes [36,37]. Both EVs and liposomes can engage CME as part of a broader repertoire of uptake routes, although its relative contribution varies depending on vesicle physicochemical properties and recipient cell type [38,39].

### 4.2. Caveolae-Mediated Endocytosis

Caveolin-mediated endocytosis represents a clathrin-independent internalization pathway driven by plasma membrane invaginations enriched in cholesterol, sphingolipids, and the scaffolding protein caveolin-1 (CAV1). Caveolae assembly and budding are orchestrated by CAV1 oligomerization and cavin proteins, while vesicle scission is largely dynamin-dependent. Upon membrane fission, caveolar vesicles are released and subsequently engage intracellular trafficking routes, including endosomal and non-degradative pathways [40,41,42].

EVs are particularly prone to caveolae-mediated uptake due to their enrichment in cholesterol and sphingomyelin, which promotes partitioning into lipid raft domains. Experimental inhibition of caveolin-1 or cholesterol depletion has been shown to reduce EV uptake in multiple cell types [27,39]. Liposomes may also enter via caveolae when formulated with rigid, cholesterol-rich membranes, although this pathway is less dominant and more formulation-dependent than for EVs.

### 4.3. Macropinocytosis

Macropinocytosis is an actin-driven uptake process initiated by small GTPases such as Rac1 and Cdc42, which promote plasma membrane ruffling and the formation of large macropinosomes [43]. Because it is not strongly constrained by particle size, macropinocytosis can efficiently internalize large extracellular cargo and nanoparticles, although this pathway frequently results in lysosomal accumulation and limited cytosolic delivery [44]. Consequently, macropinocytosis represents a highly efficient but poorly selective uptake route.

Consistent with these mechanistic features, several experimental studies have identified macropinocytosis as a major route for internalization. Using a combination of pharmacological inhibition and RNA interference approaches, Verdera et al. demonstrated that EV uptake occurs predominantly via clathrin-independent pathways, with macropinocytosis contributing substantially and clathrin-mediated endocytosis playing a negligible role. Importantly, the simultaneous inhibition of clathrin-independent endocytosis and macropinocytosis resulted in near-complete suppression of EV internalization, highlighting the independent yet complementary contributions of these pathways [45]. Notably, macropinocytosis is not exclusive to EVs. Comparative studies employing exosome-mimicking liposomes have shown that lipid composition alone can bias synthetic nanocarriers toward macropinocytic uptake, further supporting the notion that this pathway is driven primarily by vesicle physicochemical properties rather than their biological origin [46].

### 4.4. Phagocytosis

Phagocytosis is a receptor-mediated, actin-dependent uptake mechanism primarily performed by professional phagocytes. Engagement of receptors such as Fc receptors, complement receptors, or scavenger receptors triggers actin remodelling and the formation of membrane extensions that engulf the target particle, resulting in phagosome formation. Phagosomes subsequently mature through fusion with endosomes and lysosomes, leading to progressive acidification and cargo degradation [47].

In the context of EVs, phagocytosis is a major uptake pathway for immune cells. EVs displaying phosphatidylserine or opsonized by serum proteins are efficiently recognized by phagocytic receptors, leading to rapid engulfment by cells of the mononuclear phagocyte system. This process contributes significantly to EV clearance and limits their circulation time in vivo, underscoring the role of phagocytosis as a dominant sink for therapeutic EVs [48]. Experimental evidence further indicates that liposomes are similarly susceptible to phagocytic uptake. Liposome size, PEG surface density, and lipid composition were identified as key determinants of phagocytic engagement, with larger and less sterically shielded formulations undergoing more efficient uptake [49].

### 4.5. Direct Membrane Fusion

In addition to endocytosis, nanoparticles may enter cells via direct fusion with the plasma membrane. This mechanism can effectively deliver cargo into the cytosol, bypassing lysosomal degradation [38,50,51]. However, current evidence indicates that this mechanism occurs preferentially under defined physicochemical or compositional conditions and likely represents a minor uptake route compared with endocytic pathways [24].

Membrane fusion is governed by biophysical parameters such as lipid composition, membrane fluidity, and curvature stress [52]. Zhuo et al. demonstrated that membrane fusion can be selectively promoted by modulating EVs lipid composition by increasing the cholesterol content. Cholesterol-enriched vesicles fused with the plasma membrane, bypassed endosomal trafficking, and enabled direct cytosolic cargo release, as supported by molecular dynamics simulations and live-cell imaging [53].

### 4.6. Intracellular Trafficking and Fate After Uptake

Intracellular trafficking is a critical determinant of nanoparticle-mediated drug delivery, as cellular uptake alone does not guarantee effective cargo release. Most internalized nanocarriers enter the endo-lysosomal pathway, where cargo is sorted between recycling routes and degradative compartments. If endosomal escape does not occur during early trafficking stages, nanocarriers are typically delivered to lysosomes, where degradation severely limits cytosolic delivery [54,55].

For conventional liposomal formulations, the intracellular fate is frequently dominated by endosomal maturation and lysosomal accumulation, resulting in prolonged vesicular confinement and delayed or incomplete access of the encapsulated payload to the cytosolic or nuclear targets. Quantitative imaging and ultrastructural analyses of PEGylated liposomes show that the drug and liposomal membrane markers can remain highly co-localized within intracellular vesicular compartments, consistent with substantial endo-lysosomal sequestration and markedly reduced nuclear bioavailability [56].

More broadly, comparative reviews of liposomes versus EVs emphasize that, unless specifically engineered with fusogenic or stimulus-responsive features, many liposomal systems are constrained by endo-lysosomal routing, with therapeutic action often relying on slow leakage, partial carrier destabilization, or compartment-limited release rather than efficient cytosolic delivery [39]. In contrast, EVs exhibit a more heterogeneous and biologically evolved intracellular trafficking behaviour, and once internalized, do not uniformly follow the degradative endo-lysosomal pathway. Recent comparative studies suggest that certain EV populations may exhibit substantially higher apparent endosomal escape efficiencies under specific experimental conditions. These differences appear to reflect fundamental distinctions in intracellular processing rather than differences in cellular uptake alone [57].

Collectively, these observations suggest that synthetic liposomes primarily rely on engineered pH-responsive chemistries that induce relatively inefficient and stochastic endosomal escape. In contrast, EVs exploit biologically evolved trafficking pathways and membrane interaction mechanisms that, in certain contexts, can enable more efficient cytosolic cargo delivery with less reliance on nonspecific endosomal disruption.

## 5. Comparison of EVs and Liposomes in Oncology

Only a limited number of studies have performed direct head-to-head comparisons between EVs and synthetic liposomes in oncology (Table 1), and the available evidence remains highly heterogeneous in terms of tumor models, EV cellular sources, therapeutic payloads, administration strategies, and experimental endpoints. A direct comparison between naturally derived EVs and synthetic liposomes was reported by Conlon T. et al., comparing nanoalgosomes, isolated from the microalgae Tetraselmis chuii, with liposomes loaded with pirfenidone and quercetin, two drugs commonly used for the treatment of pulmonary fibrosis [58]. Unlike synthetic liposomes, nanoalgosomes represent naturally occurring EVs endowed with intrinsic bioactive properties, including anti-inflammatory and antioxidant activity [59]. Using bleomycin-induced stress in human lung adenocarcinoma alveolar epithelial cells (A549), the study evaluated the impact of the two nanocarrier systems on cellular migration, oxidative stress, epithelial–mesenchymal transition (EMT), and Transforming Growth Factor-β1 (TGF-β1) expression. Both nanoalgosomes and drug-loaded liposomes significantly reduced intracellular reactive oxygen species (ROS) levels and inhibited cell migration in a dose-dependent manner compared with the bleomycin-treated control group. In addition, treatment with both vesicle types resulted in downregulation of the mesenchymal markers TGF-β1 and vimentin (VIM) and upregulation of the epithelial marker CDH1, suggesting a partial reversal of EMT-associated transcriptional programs [58]. Overall, these findings indicate that naturally derived EVs and synthetic liposomes can exert comparable modulatory effects on oxidative stress and EMT-related signalling pathways, highlighting the potential of naturally derived vesicles as alternative nanocarriers for respiratory diseases and cancer therapy.

A study of Kamerkar S. et al. evaluated the therapeutic performance of engineered exosomes (iExosomes) and synthetic liposomes (iLiposomes) loaded with siRNA targeting KRASG12D, one of the most prevalent oncogenic mutations in pancreatic ductal adenocarcinoma (PDAC). Exosomes were isolated from normal human foreskin fibroblasts (BJ cells), while liposomes were synthetically produced and loaded with the same siRNA payload. In an orthotopic PANC-1 mouse model, iExosomes displayed a markedly superior capacity to deliver KRAS-targeting siRNA and suppress tumor growth compared with liposomes. After 30 days of treatment, mice receiving iExosomes showed a significant reduction in metastatic burden and improved survival, whereas iLiposomes produced only a moderate inhibition of tumor growth. The enhanced therapeutic performance of EVs was attributed to their plasma-membrane-like lipid composition and the presence of membrane proteins such as CD47, which may reduce clearance by the mononuclear phagocyte system and prolong circulation time. In addition, these biological features likely contribute to efficient EV uptake by KRAS-mutant pancreatic cancer cells despite the dense stromal architecture characteristic of PDAC tumors [60].

A further comparison has been reported in glioma models. Gliomas represent the most common and aggressive primary tumors of the central nervous system, characterized by high genetic heterogeneity and resistance to conventional therapies. In this context, ferroptosis-inducing therapies have emerged as a promising strategy to eliminate therapy-resistant cancer cells. Hao W. et al. compared the anticancer activity of natural killer cell-derived EVs (NK-EVs) with that of RSL3-loaded liposomes (RLPs), where RSL3 acts as a GPX4 inhibitor capable of inducing ferroptosis. In vitro analyses showed that NK-EVs produced greater cytotoxicity and higher apoptotic rates (47.2%) compared with RLPs (28.4%), indicating stronger intrinsic antitumor activity. Importantly, NK-EVs also induced the maturation of bone marrow-derived dendritic cells toward a CD86^+^/CD80^+^ immunostimulatory phenotype, accompanied by increased secretion of pro-inflammatory cytokines such as TNF-α and IL-6. Conversely, RLPs primarily promoted intracellular accumulation of reactive oxygen species and lipid peroxides, consistent with their ferroptosis-inducing mechanism. In vivo studies in glioma-bearing mice further demonstrated higher accumulation and prolonged retention of NK-EVs in brain tumor tissues compared with liposomes, supporting the hypothesis that EVs retain tumor-homing features inherited from their parental immune cells [61].

In the context of chemo-immunotherapy, Zhu T. et al. investigated paclitaxel delivery using exosomes derived from bispecific CAR-T cells targeting mesothelin (MSLN) and PD-L1 (CAR-T-Exo@PTX) and lung-targeted liposomes loaded with paclitaxel (Liposome@PTX). In vitro experiments demonstrated that CAR-T-Exo@PTX induced stronger dendritic cell maturation and higher secretion of the pro-inflammatory cytokine TNF-α compared with Liposome@PTX, highlighting the immunomodulatory properties inherited from the parental CAR-T cells. However, in vivo pharmacokinetic analyses revealed a different trend. Liposome@PTX achieved significantly higher paclitaxel accumulation in lung tissues and improved overall survival in tumor-bearing mice (42 days) compared with CAR-T-Exo@PTX (34 days). These findings indicate that liposomal formulations may retain advantages in drug loading capacity and controlled drug release, whereas EV-based systems may contribute more prominently to immune activation and biological targeting mechanisms [62].

Taken together, these comparative studies suggest that EVs and liposomes display complementary advantages rather than universally superior performance of one platform over the other. The currently available preclinical evidence suggests that EVs may provide advantages in biological targeting, immune modulation, and tissue penetration, whereas liposomes retain superior manufacturing reproducibility, loading efficiency, and clinical scalability.

Nevertheless, the number of studies performing direct head-to-head comparisons between EVs and liposomes within the same tumor model remains limited. Existing comparisons differ substantially with respect to tumor type, EV origin, therapeutic cargo, administration routes, and evaluated biological endpoints. Consequently, direct cross-study comparisons are difficult, and it remains unclear whether one platform consistently outperforms the other across different oncological settings. This lack of standardized comparative studies represents a major knowledge gap in the field and highlights the need for systematic side-by-side evaluations performed under harmonized experimental conditions. In response to these limitations, recent research has increasingly focused on hybrid nanocarriers designed to integrate the advantages of both platforms.

## 6. Hybrid EV–Liposome Nanoparticles

**Figure 3:**
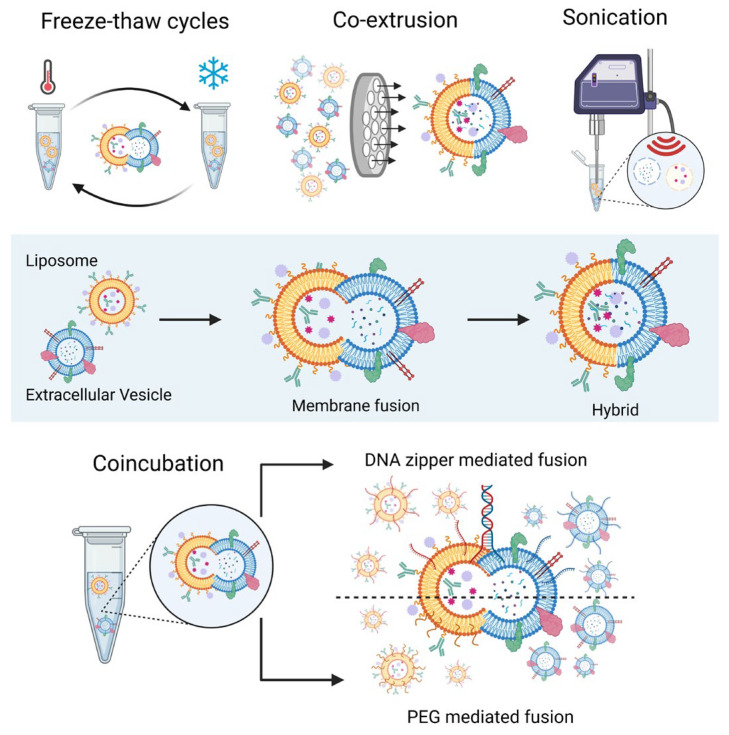
Schematic representation of the main strategies used to generate HELNs. High-energy approaches such as freeze–thaw cycling, co-extrusion, and ultrasonication promote membrane fusion through transient membrane destabilization and generally provide relatively high fusion efficiency and homogeneous vesicle populations. However, these methods may also induce membrane damage, cargo leakage, vesicle aggregation, or partial alteration of EV membrane proteins and biological functionality due to mechanical or thermal stress. In contrast, minimally disruptive approaches including co-incubation, PEG-mediated fusion, and DNA zipper-mediated fusion rely on spontaneous or controlled membrane interactions under milder conditions, thereby better preserving EV membrane composition, receptor orientation, and targeting properties. Nevertheless, these strategies may exhibit lower fusion efficiency, reduced scalability, or increased heterogeneity of the resulting hybrid vesicles. Overall, the choice of hybridization method represents a critical balance between fusion efficiency, structural stability, scalability, and preservation of EV biological activity.

Several strategies have been developed to generate hybrid EV-liposome nanoparticles (HELNs) through membrane fusion between EVs and liposomes (Figure 3) [63,64]. Among the earliest and most widely used approaches is freeze–thaw cycling, in which repeated freezing and thawing transiently disrupt vesicle membranes and promote their fusion. Although technically simple and widely applicable, repeated freeze–thaw cycles may partially alter EV membrane integrity, induce vesicle aggregation, and affect the conformation or orientation of membrane-associated proteins involved in biological targeting. Co-extrusion methods represent another commonly used strategy, where EVs and liposomes are forced through polycarbonate membranes; the resulting mechanical shear and pressure facilitate membrane integration and produce hybrid vesicles with relatively uniform size distributions [64]. However, the high mechanical stress generated during extrusion may also contribute to partial membrane remodelling and modification of surface protein organization, potentially influencing the biological functionality of EV-derived membranes.

Extrusion procedures can also be combined with ultrasonication, which induces transient membrane destabilization and further promotes vesicle fusion [65]. Despite their efficiency in promoting hybridization, ultrasonication-based approaches may increase the risk of membrane damage, cargo leakage, protein denaturation, and alteration of native EV surface architecture due to local thermal and mechanical stress.

In addition to these mechanically driven approaches, several minimally disruptive strategies have been developed to better preserve membrane proteins and cargo integrity during hybridization. These include simple co-incubation, where spontaneous membrane fusion occurs under physiological conditions and is particularly efficient with cationic liposomes [66], PEG-mediated fusion, in which polyethylene glycol reduces electrostatic repulsion between vesicles [63], and DNA zipper-mediated fusion, where complementary DNA strands tether vesicles and promote controlled membrane fusion [67]. Compared with high-energy fusion methods, these milder approaches are generally considered more suitable for preserving EV membrane composition, receptor orientation, and biological targeting functionality, although fusion efficiency may be lower and hybrid populations more heterogeneous.

Recent studies have explored HELNs for a wide range of oncological applications (Table 2) [68]. Compared with conventional liposomes, HELNs loaded with chemotherapeutic agents frequently exhibit improved pharmacokinetics and enhanced antitumor efficacy, largely due to improved tumor targeting and prolonged circulation time [69,70,71,72,73,74,75,76,77,78,79,80,81,82,83]. Importantly, these improvements are often accompanied by reduced systemic toxicity, highlighting the potential translational advantages of hybrid systems [73,74].

The biological origin of the EV component strongly influences HELN functionality. Hybrid vesicles incorporating tumor-derived EVs can exploit homologous targeting mechanisms, facilitating preferential accumulation within tumours derived from the same cell lineage [73,77,78,80]. Conversely, HELNs based on immune-cell-derived EVs may exert immunomodulatory effects, including the induction of immunogenic cell death, modulation of the tumor microenvironment, and enhanced T-cell activation [61,62,76,82,83].

Beyond conventional chemotherapy delivery, HELNs have also demonstrated promise in gene therapy applications. Hybrid vesicles can partially overcome the limited nucleic acid loading capacity and structural instability typically associated with native EVs, enabling efficient delivery of siRNA [78], miRNA [81,84], mRNA [85], and CRISPR/Cas systems [75].

HELNs have also been explored in stimuli-responsive therapeutic strategies, including photothermal therapy. By integrating EV membranes with thermosensitive or photoresponsive liposomes, hybrid systems can remain stable under physiological conditions but release their cargo rapidly upon near-infrared irradiation, enabling spatiotemporally controlled drug release [76,80,82,86,87].

In addition to therapeutic applications, HELNs are emerging as promising platforms for tumor imaging and precision diagnostics. Their prolonged circulation time, targeting ability, and capacity to cross biological barriers make them attractive carriers for imaging probes, enabling real-time therapeutic monitoring and image-guided therapy. Furthermore, hybrid fusion strategies have been adapted for in situ detection of EV-associated biomarkers, particularly miRNAs thereby improving diagnostic sensitivity and workflow efficiency [84,86,88].

Overall, hybrid EV–liposome nanoparticles represent a versatile nanoplatform that integrates biomimetic and synthetic design principles. By combining the engineering versatility of liposomes with the biological functionality of EV membranes, HELNs may help overcome key limitations of both delivery systems and represent a promising strategy for next-generation nanomedicine approaches in cancer therapy.

**Table 2:** Effects of different types of HELNs on tumor cell growth in vitro and in vivo.

Tumor Type	Therapy	EV Source	Hybridization Procedure	Effects of Hybrid	Ref.
Glioblastoma	photothermal and gene therapy	Macrophages	coincubation liposomes + EV + PEG8000	Reduction in tumor growth and increased survival	[79]
	photothermal therapy	M1 murine macrophage cell line	membrane extrusion	Increased cellular uptake and cytotoxicity, tumor volume reduction and survival	[86]
Melanoma	photothermal therapy and immunomodulation	M1 macrophage cell line	membrane fusion (freeze–thaw method)	Reduction in tumor growth, prolonged survival, and stronger anti-tumor immune response	[82]
	vaccination	cell lines and bacteria as adjuvant	sonication with a probe, then coextrusion	Increased dendritic cell maturation and in vivo reduction in tumor growth	[83]
	tumor targeting	M1 or M0 murine macrophages	membrane fusion (freeze–thaw method)	Increased thermoresponsiveness	[87]
	capture of circulating tumor cells	murine melanoma cells	membrane fusion and extrusion	Increased uptake and efficient capture cells from blood	[88]
Ovarian cancer	miRNA therapy and immunotherapy	CD47^+^ cisplatin-resistant ovarian carcinoma cell line	membrane fusion through sonication and extrusion	Increased stability and uptake, induction of M2 to M1 polarization and reduction in tumor volume	[81]
Brest cancer	tumor microenvironment targeting and photothermal therapy	M1-like macrophages	coextruded	Enhanced intracellular delivery and cytotoxicity, in vivo reduction in tumor	[76]
	tumor targeting	breast cancer cells	liposome film hydrated with EVs then serial extrusion	Enhanced internalization and production of inflammatory cytokines	[77]
Pancreatic ductal adenocarcinoma	chemotherapy	carcinoma cell line	sonication and extrusion	Enhanced tumor cell uptake, apoptosis induction and migration block	[70]
Colon cancer	chemotherapy and immunomodulation	MSCs	freeze–thaw	Increased cellular uptakes and apoptosis and in vivo reduction in tumor growth with increased survival	[72]
	chemotherapy	colon cancer cell line	coextrusion	Higher cellular uptake and toxicity and in vivo suppression of tumor growth	[73]
	photothermal therapy and immunomodulation	CD47^+^ CT26 coloncarcinoma cell line	membrane fusion (freeze–thaw method)	Better drug release, cellular uptake, induction of apoptosis, dendritic cell maturation, in vivo reduction in tumor growth and prolonged survival	[80]
	chemotherapy and immunomodulation	Car-T cells	film hydration and extrusion	Induction of tumor cell cytotoxicity, dendritic cell maturation and in vivo suppression of tumor growth and prolonged the survival time	[62]
	gene therapy	293T	freeze–thaw method	Reduction in tumor size	[85]
Solid tumors	immunomodulation	B16F10, BL6, CT26, and GL261 cells	freeze–thaw and sonication	Reduction in tumor growth	[78]
Glioma	ferroptosis and immunomodulation	Natural Killer Cells	coincubation of Liposome, NK-EVs, and PEG8000	Enhanced cellular uptake, apoptosis, lipid peroxidation, dendritic cell maturation and in vivo reduction in tumor growth and increased survival.	[61]

## 7. Translational and Regulatory Challenges for Clinical Implementation

Despite major advances in liposomes, EVs, and HELNs, clinical translation remains limited by manufacturing, regulatory, and economic barriers that extend beyond biological efficacy. These challenges vary significantly among platforms due to differences in biological complexity and engineering controllability.

### 7.1. Manufacturing and Good Manufacturing Practice (GMP) Scalability

Liposomes represent the most clinically mature platform, supported by well-established and scalable manufacturing methods (e.g., thin-film hydration, microfluidics, ethanol injection) and robust GMP production with high batch-to-batch reproducibility and multiple regulatory approvals [5,11,15].

In contrast, EV manufacturing remains a major limitation. Production is strongly influenced by donor cell source and culture conditions, leading to intrinsic heterogeneity and poor batch consistency. In addition, isolation techniques (ultracentrifugation, size-exclusion chromatography, ultrafiltration, immunoaffinity capture) involve trade-offs between purity, yield, scalability, and cost, hindering large-scale standardization [7,8,89,90,91].

HELNs further increase production complexity, as hybridization requires controlled membrane fusion while preserving EV integrity and cargo distribution. Variability in fusion efficiency and product heterogeneity currently limits reproducibility and industrial scalability.

### 7.2. Quality Control and Characterization

Liposome characterization is well standardized, including size distribution, polydispersity, zeta potential, encapsulation efficiency, drug release, and stability.

EVs require more complex and less standardized characterization, combining physical and molecular markers. However, no consensus exists regarding potency assays or the correlation between EV properties and biological activity.

HELNs require dual characterization of both synthetic and biological components, including fusion efficiency, cargo loading, membrane integrity, and stability, highlighting the absence of unified analytical standards.

### 7.3. Regulatory Landscape and Economic Considerations

Liposomes benefit from established regulatory frameworks and clear approval pathways. By contrast, EV-based therapeutics are currently evaluated case-by-case, with unresolved issues including donor eligibility, safety assessment, biodistribution, and product consistency. HELNs lack defined regulatory classification, as they combine features of biologics and nanomedicines. Dedicated regulatory guidelines will likely be required to address their hybrid nature.

Moreover, liposomal products benefit from mature industrial processes and relatively predictable production costs. EVs remain expensive and labor-intensive, particularly under GMP conditions. HELNs may further increase manufacturing costs due to additional hybridization steps and process complexity. Future clinical translation will depend not only on therapeutic performance but also on scalable manufacturing, standardized characterization, and cost-effective production strategies.

Overall, liposomes remain the most clinically advanced platform, whereas EVs and HELNs offer superior biological functionality but face significant barriers in scalability, standardization, regulatory definition, and cost-efficiency. A comparative overview of the main translational and technological features of liposomes, EVs, and HELNs is provided in Table 3.

## 8. Conclusions and Future Perspectives

The development of nanoscale delivery systems has considerably expanded the therapeutic landscape of cancer treatment. Among these platforms, liposomes and EVs represent two of the most extensively investigated nanocarriers, each providing distinct advantages for drug delivery applications. Although direct comparative studies between EVs and liposomes remain limited, current evidence suggests that these systems should be considered complementary rather than competing technologies [58,59,60,61,62]. EVs display biologically evolved targeting and immunomodulatory capabilities, whereas liposomes retain important advantages in formulation reproducibility, drug encapsulation efficiency, scalable manufacturing, and regulatory maturity. This complementarity has driven the development of HELNs, designed to integrate the biomimetic properties of EV membranes with the engineering flexibility of synthetic nanocarriers [69,70,71,72,73,74,75,76,77,78,79,80,81,82,83,84,85,86,87,88].

Recent advances in HELN engineering indicate that these hybrid systems may improve tumor targeting, drug loading, controlled release, and delivery of complex therapeutic payloads, including nucleic acids and genome-editing tools. In addition, their potential applications extend beyond drug delivery to cancer immunotherapy and molecular imaging. Despite these promising characteristics, HELNs remain predominantly at the preclinical stage and have not yet entered clinical trials.

**Figure 4:**
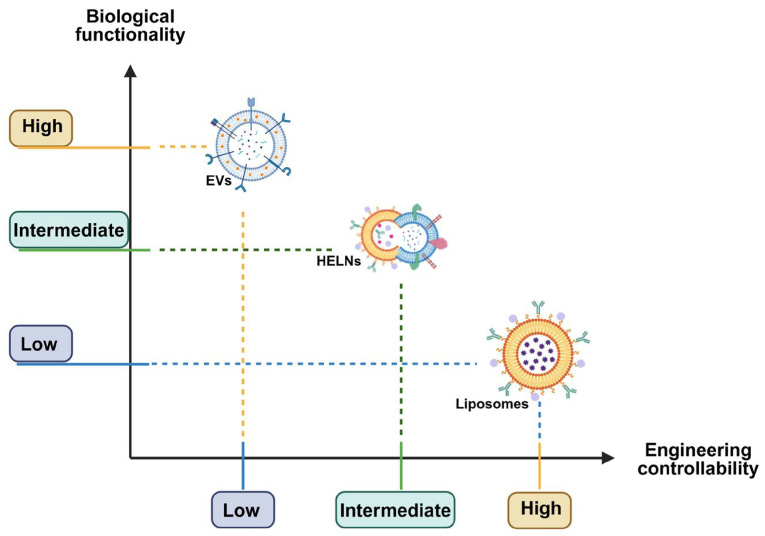
Conceptual framework positioning liposomes, EVs, and HELNs according to biological functionality and engineering controllability.

In summary, liposomes provide the highest degree of manufacturing reproducibility, formulation control, and regulatory maturity, whereas EVs exhibit superior biological targeting and intercellular communication properties. HELNs occupy an intermediate position, aiming to combine the engineering advantages of synthetic nanocarriers with the biological functionality of naturally derived vesicles (Figure 4).

A major unmet need is the implementation of systematic head-to-head comparative studies between EVs, liposomes, and HELNs under standardized experimental conditions. Current investigations remain highly heterogeneous in terms of tumor models, EV cellular sources, therapeutic payloads, administration routes, and evaluated endpoints, limiting definitive conclusions regarding the relative advantages of each platform. Future studies should therefore employ clinically relevant models, including patient-derived xenografts and immunocompetent systems, together with comprehensive biodistribution and pharmacokinetic analyses to better characterize nanoparticle circulation, tissue accumulation, intracellular trafficking, and long-term safety profiles in vivo.

Overall, current evidence does not support the conclusion that either EVs or liposomes are universally superior nanocarriers for oncological applications. Rather, these systems appear to possess complementary biological and translational properties. In this context, HELNs may represent one of the most promising future directions in precision nanomedicine, potentially combining the controllability and reproducibility of synthetic systems with the biological functionality and targeting capabilities of naturally derived EVs. However, whether HELNs will ultimately overcome the translational limitations associated with both parent platforms remains to be demonstrated through rigorous preclinical and clinical investigations.

The convergence of synthetic nanotechnology and biologically inspired EV systems is opening new opportunities for the development of next-generation drug delivery platforms. By integrating the precision engineering of liposomes with the biological complexity of EV membranes, HELNs may contribute to the development of more efficient, targeted, and personalized cancer therapies, ultimately advancing precision nanomedicine in oncology.

## Figures and Tables

**Table 1:** Direct comparison of the therapeutic efficacy of EVs and liposomes in oncology.

Tumor Type	EV Source	Liposome Formulation	Comparative Effects of Liposomes and EVs	Ref.
Lung adenocarcinoma	Microalgae Tetraselmis chuii	Synthetic liposomes loaded with pirfenidone and quercetin	Comparable effects of EVs and liposomes on reduction in ROS, inhibition of cell migration, downregulation of mesenchymal markers, upregulation of epithelial markers	[58]
Pancreatic tumor	Human foreskin fibroblasts (Bj cells); loaded with siRNA targeting KRASG12D	Synthetic liposomes loaded with siRNA targeting KRASG12D	EVs: superior capacity to suppress tumor growth and improve survival in vivo	[60]
Glioma	Natural killer cells	RSL3-loaded liposomes	EVs: stronger antitumor activity, induction of dendritic cells maturation and higher accumulation in vivo. Liposomes: higher intracellular accumulation of ROS and lipid peroxides	[61]
Lung cancer	Bispecific CAR-T cells	Lung-targeted liposomes loaded with paclitaxel	EVs: stronger dendritic cell maturation and higher secretion of pro-inflammatory cytokines Liposomes: improved overall survival and drug release in vivo	[62]

**Table 3:** Comparative overview of liposomes, EVs, and HELNs in terms of manufacturing, biological, regulatory, and translational features.

Parameters	Liposomes	EVs	HELNs
Manufacturing scalability	High	Low–Moderate	Currently low
Drug loading efficiency	High	Moderate	Moderate–High
Biological targeting	Limited	High	High
Batch reproducibility	High	Low	Intermediate
Clinical approvals	Multiple	None	None
Immunogenicity	Low–Moderate	Low	Unknown
Endosomal escape	Limited	Potentially higher	Under investigation
GMP manufacturing readiness	High	Emerging	Early stage

## Data Availability

No new data were created or analyzed in this study. Data sharing is not applicable to this article.

## References

[1] Guzowska J., Kowalski S., Schachta I., Piekuś-Słomka N., Słomka A. (2026). Connecting the Dots: Milestones in the History of Extracellular Vesicle Research. Int. J. Mol. Sci..

[2] Fraczek W., Szmidt M., Kregielewski K., Grodzik M. (2025). Liposomes and extracellular vesicles as distinct paths toward precision glioma treatment. Int. J. Mol. Sci..

[3] Allen T.M., Cullis P.R. (2013). Liposomal drug delivery systems: From concept to clinical applications. Adv. Drug Deliv. Rev..

[4] Bozzuto G., Molinari A. (2015). Liposomes as nanomedical devices. Int. J. Nanomed..

[5] Pattni B.S., Chupin V.V., Torchilin V.P. (2015). New developments in liposomal drug delivery. Chem. Rev..

[6] Al-Jipouri A., Almurisi S.H., Al-Japairai K., Bakar L.M., Doolaanea A.A. (2023). Liposomes or extracellular vesicles: A comprehensive comparison of both lipid bilayer vesicles for pulmonary drug delivery. Polymers.

[7] Vader P., Mol E.A., Pasterkamp G., Schiffelers R.M. (2016). Extracellular vesicles for drug delivery. Adv. Drug Deliv. Rev..

[8] Piffoux M., Nicolás-Boluda A., Mulens-Arias V., Richard S., Rahmi G., Gazeau F., Wilhelm C., Silva A.K.A. (2019). Extracellular vesicles for personalized medicine: The input of physically triggered production, loading and theranostic properties. Adv. Drug Deliv. Rev..

[9] Karmacharya M., Kumar S., Cho Y.K. (2023). Tuning the Extracellular Vesicles Membrane through Fusion for Biomedical Applications. J. Funct. Biomater..

[10] Liu A., Yang G., Liu Y., Liu T. (2022). Research progress in membrane fusion-based hybrid exosomes for drug delivery systems. Front. Bioeng. Biotechnol..

[11] Torchilin V.P. (2005). Recent advances with liposomes as pharmaceutical carriers. Nat. Rev. Drug Discov..

[12] Michael A., Bhattacharya D., Gopal V., Suresh D., Arun J., Ganesh G.N.K. (2026). Liposomes as versatile drug delivery vehicles: Emerging trends, technological innovations and future perspectives. J. Liposome Res..

[13] Vagena I.-A., Malapani C., Gatou M.-A., Lagopati N., Pavlatou E.A. (2025). Enhancement of EPR effect for passive tumor targeting: Current status and future perspectives. Appl. Sci..

[14] Khan M.S., Alqahtani T., Al Shmrany H., Gupta G., Goh K.W., Sahebkar A., Kesharwani P. (2026). Enhanced permeability and retention effect: Advances in nanomedicine for improved tumor targeting. Biomater. Adv..

[15] Chen J., Hu S., Sun M., Shi J., Zhang H., Yu H., Yang Z. (2024). Recent advances and clinical translation of liposomal delivery systems in cancer therapy. Eur. J. Pharm. Sci..

[16] Gabizon A.A., Shmeeda H., Zalipsky S. (2006). Pros and cons of the liposome platform in cancer drug targeting. J. Liposome Res..

[17] Cheng Z., Huang H., Yin M., Liu H. (2025). Applications of liposomes and lipid nanoparticles in cancer therapy: Current advances and prospects. Exp. Hematol. Oncol..

[18] Jain R.K., Stylianopoulos T. (2010). Delivering nanomedicine to solid tumors. Nat. Rev. Clin. Oncol..

[19] Blanco E., Shen H., Ferrari M. (2015). Principles of nanoparticle design for overcoming biological barriers to drug delivery. Nat. Biotechnol..

[20] Wilhelm S., Tavares A., Dai Q., Ohta S., Audet J., Dvorak H.F., Chan W.C.W. (2016). Analysis of nanoparticle delivery to tumours. Nat. Rev. Mater..

[21] Mohamed M., Abu Lila A.S., Shimizu T., Alaaeldin E., Hussein A., Sarhan H.A., Szebeni J., Ishida T. (2019). PEGylated liposomes: Immunological responses. Sci. Technol. Adv. Mater..

[22] Yáñez-Mó M., Siljander P.R.M., Andreu Z., Zavec A.B., Borràs F.E., Buzas E.I., Buzas K., Casal E., Cappello F., Carvalho J. (2015). Biological properties of extracellular vesicles and their physiological functions. J. Extracell. Vesicles.

[23] van Niel G., D’Angelo G., Raposo G. (2018). Shedding light on the cell biology of extracellular vesicles. Nat. Rev. Mol. Cell Biol..

[24] Mathieu M., Martin-Jaular L., Lavieu G., Théry C. (2019). Specificities of secretion and uptake of exosomes and other extracellular vesicles for cell-to-cell communication. Nat. Cell Biol..

[25] Greening D.W., Xu R., Rai A., Suwakulsiri W., Chen M., Simpson R.J. (2025). Clinical relevance of extracellular vesicles in cancer—Therapeutic and diagnostic potential. Nat. Rev. Clin. Oncol..

[26] van der Koog L., Gandek T.B., Nagelkerke A. (2022). Liposomes and extracellular vesicles as drug delivery systems: A comparison of composition, pharmacokinetics, and functionalization. Adv. Healthc. Mater..

[27] Jackson Cullison S.R., Flemming J.P., Karagoz K., Wermuth P.J., Mahoney M.G. (2024). Mechanisms of extracellular vesicle uptake and implications for the design of cancer therapeutics. J. Extracell. Biol..

[28] Bader J., Brigger F., Leroux J.-C. (2024). Extracellular vesicles versus lipid nanoparticles for the delivery of nucleic acids. Adv. Drug Deliv. Rev..

[29] Lu X., Fan S., Cao M., Liu D., Xuan K., Liu A. (2024). Extracellular vesicles as drug delivery systems in therapeutics: Current strategies and future challenges. J. Pharm. Investig..

[30] Rennick J.J., Johnston A.P.R., Parton R.G. (2021). Key principles and methods for studying the endocytosis of biological and nanoparticle therapeutics. Nat. Nanotechnol..

[31] Ribovski L., Joshi B., Gao J., Zuhorn I. (2023). Breaking free: Endocytosis and endosomal escape of extracellular vesicles. Extracell. Vesicles Circ. Nucl. Acids.

[32] Digiacomo L., Cardarelli F., Pozzi D., Palchetti S., Digman M.A., Gratton E., Capriotti A.L., Mahmoudi M., Caracciolo G. (2017). An apolipoprotein-enriched biomolecular corona switches the cellular uptake mechanism and trafficking pathway of lipid nanoparticles. Nanoscale.

[33] Tóth E.Á., Turiák L., Visnovitz T., Cserép C., Mázló A., Sódar B.W., Försönits A.I., Petővári G., Sebestyén A., Komlósi Z. (2021). Formation of a protein corona on the surface of extracellular vesicles in blood plasma. J. Extracell. Vesicles.

[34] Voke E., Arral M.L., Squire H.J., Lin T.J., Zheng L., Coreas R., Lui A., Iavarone A.T., Pinals R.L., Whitehead K.A. (2025). Protein corona formed on lipid nanoparticles compromises delivery efficiency of mRNA cargo. Nat. Commun..

[35] Roth T.F., Porter K.R. (1964). Yolk protein uptake in the oocyte of the mosquito *Aedes aegypti*. J. Cell Biol..

[36] McMahon H.T., Boucrot E. (2011). Molecular mechanism and physiological functions of clathrin-mediated endocytosis. Nat. Rev. Mol. Cell Biol..

[37] Ehrlich M., Boll W., van Oijen A., Hariharan R., Chandran K., Nibert M.L., Kirchhausen T. (2004). Endocytosis by random initiation and stabilization of clathrin-coated pits. Cell.

[38] Mulcahy L.A., Pink R.C., Carter D.R.F. (2014). Routes and mechanisms of extracellular vesicle uptake. J. Extracell. Vesicles.

[39] Gandek T.B., van der Koog L., Nagelkerke A. (2023). A comparison of cellular uptake mechanisms, delivery efficacy, and intracellular fate between liposomes and extracellular vesicles. Adv. Healthc. Mater..

[40] Matthaeus C., Sochacki K.A., Dickey A.M., Puchkov D., Haucke V., Lehmann M., Taraska J.W. (2022). The molecular organization of differentially curved caveolae indicates bendable structural units at the plasma membrane. Nat. Commun..

[41] Stea D.M., D’Alessio A. (2025). Caveolae: Metabolic platforms at the crossroads of health and disease. Int. J. Mol. Sci..

[42] D’Alessio A. (2023). Unraveling the cave: A seventy-year journey into the caveolar network, cellular signaling, and human disease. Cells.

[43] Swanson J.A., Yoshida S. (2019). Macropinosomes as units of signal transduction. Philos. Trans. R. Soc. B Biol. Sci..

[44] Means N., Elechalawar C.K., Chen W.R., Bhattacharya R., Mukherjee P. (2022). Revealing macropinocytosis using nanoparticles. Mol. Asp. Med..

[45] Costa Verdera H., Gitz-Francois J.J., Schiffelers R.M., Vader P. (2017). Cellular uptake of extracellular vesicles is mediated by clathrin-independent endocytosis and macropinocytosis. J. Control. Release.

[46] Lu M., Zhao X., Xing H., Xun Z., Zhu S., Lang L., Yang T., Cai C., Wang D., Ding P. (2018). Comparison of exosome-mimicking liposomes with conventional liposomes for intracellular delivery of siRNA. Int. J. Pharm..

[47] Freeman S.A., Grinstein S. (2014). Phagocytosis: Receptors, signal integration, and the cytoskeleton. Immunol. Rev..

[48] Mylvaganam S., Freeman S.A., Grinstein S. (2021). The cytoskeleton in phagocytosis and macropinocytosis. Curr. Biol..

[49] Giambelluca M., Markova E., Louet C., Steinkjer B., Sundset R., Škalko-Basnet N., Hak S. (2023). Liposomes–human phagocytes interplay in whole blood: Effect of liposome design. Nanomedicine.

[50] Kim H.I., Park J., Zhu Y., Wang X., Han Y., Zhang D. (2024). Recent advances in extracellular vesicles for therapeutic cargo delivery. Exp. Mol. Med..

[51] Paramshetti S., Angolkar M., Talath S., Osmani R.A.M., Spandana A., Al Fatease A., Hani U., Ramesh K.V.R.N.S., Singh E. (2024). Unravelling the in vivo dynamics of liposomes: Insights into biodistribution and cellular membrane interactions. Life Sci..

[52] Joardar A., Pattnaik G.P., Chakraborty H. (2022). Mechanism of membrane fusion: Interplay of lipid and peptide. J. Membr. Biol..

[53] Zhuo Y., Luo Z., Zhu Z., Wang J., Li X., Zhang Z., Guo C., Wang B., Nie D., Gan Y. (2024). Direct cytosolic delivery of siRNA via cell membrane fusion using cholesterol-enriched exosomes. Nat. Nanotechnol..

[54] Winkeljann B., Keul D.C., Merkel O.M. (2023). Engineering poly- and micelleplexes for nucleic acid delivery—A reflection on their endosomal escape. J. Control. Release.

[55] Ahmad A., Khan J.M., Paray B.A., Rashid K., Parvez A. (2024). Endolysosomal trapping of therapeutics and endosomal escape strategies. Drug Discov. Today.

[56] Seynhaeve A.L.B., Dicheva B.M., Hoving S., Koning G.A., Ten Hagen T.L.M. (2013). Intact Doxil is taken up intracellularly and released doxorubicin sequesters in the lysosome: Evaluated by in vitro/in vivo live cell imaging. J. Control. Release.

[57] Hagedorn L., Jürgens D.C., Merkel O.M., Winkeljann B. (2024). Endosomal escape mechanisms of extracellular vesicle-based drug carriers: Lessons for lipid nanoparticle design. Extracell. Vesicles Circ. Nucl. Acids.

[58] Conlon T., Schaaf M., Mateos-Maroto A., Picciotto S., Morsbach S., Adamo G., Si S., Lieberwirth I., Rosenauer C., Landfester K. (2025). Comparative effects of extracellular vesicles and liposomal nanocarriers on bleomycin-induced stress in A549 human adenocarcinoma cells. Biomed. Pharmacother..

[59] Adamo G., Santonicola P., Picciotto S., Gargano P., Nicosia A., Longo V., Aloi N., Romancino D.P., Paterna A., Rao E. (2024). Extracellular vesicles from the microalga *Tetraselmis chuii* are biocompatible and exhibit unique bone tropism along with antioxidant and anti-inflammatory properties. Commun. Biol..

[60] Kamerkar S., LeBleu V.S., Sugimoto H., Yang S., Ruivo C.F., Melo S.A., Lee J.J., Kalluri R. (2017). Exosomes facilitate therapeutic targeting of oncogenic KRAS in pancreatic cancer. Nature.

[61] Hao W., Sun N., Fan Y., Chen M., Liu Q., Yang M., Yang Y., Gao C. (2024). Targeted ferroptosis-immunotherapy synergy: Enhanced antiglioma efficacy with hybrid nanovesicles comprising NK cell-derived exosomes and RSL3-loaded liposomes. ACS Appl. Mater. Interfaces.

[62] Zhu T., Chen Z., Jiang G., Huang X. (2023). Sequential targeting hybrid nanovesicles composed of chimeric antigen receptor T-cell-derived exosomes and liposomes for enhanced cancer immunochemotherapy. ACS Nano.

[63] Piffoux M., Silva A.K.A., Wilhelm C., Gazeau F., Tareste D. (2018). Modification of extracellular vesicles by fusion with liposomes for the design of personalized biogenic drug delivery systems. ACS Nano.

[64] Moholkar D.N., Kandimalla R., Gupta R.C., Aqil F. (2023). Advances in lipid-based carriers for cancer therapeutics: Liposomes, exosomes and hybrid exosomes. Cancer Lett..

[65] Jhan Y.-Y., Prasca-Chamorro D., Palou Zuniga G., Moore D.M., Arun Kumar S., Gaharwar A.K., Bishop C.J. (2020). Engineered extracellular vesicles with synthetic lipids via membrane fusion to establish efficient gene delivery. Int. J. Pharm..

[66] Lin Y., Wu J., Gu W., Huang Y., Tong Z., Huang L., Tan J. (2018). Exosome-liposome hybrid nanoparticles deliver CRISPR/Cas9 system in MSCs. Adv. Sci..

[67] Malle M.G., Song P., Löffler P.M.G., Kalisi N., Yan Y., Valero J., Vogel S., Kjems J. (2024). Programmable RNA loading of extracellular vesicles with toehold-release purification. J. Am. Chem. Soc..

[68] Dou J., Wang J., Zhang G., Fu X., Zhang Y., Sun F. (2026). Advances in hybrid exosome-liposome nanoparticles for enhanced cancer therapy. Colloids Surf. B Biointerfaces.

[69] Qiao S., Hu S., Huang K., Su T., Li Z., Vandergriff A., Cores J., Dinh P.-U., Allen T., Shen D. (2020). Tumor cell-derived exosomes home to their cells of origin and can be used as Trojan horses to deliver cancer drugs. Theranostics.

[70] Kim H., Park H., Liu H., Kim S., Lee Y., Kim Y.-C. (2024). Hybrid nanoparticles of extracellular vesicles and gemcitabine prodrug-loaded liposomes with enhanced targeting ability for effective PDAC treatment. ACS Appl. Bio Mater..

[71] Tiwari R.P., Shukla K., Yadav M., Sharma A.K., Bakshi D., Panwar N., Singh N., Agarwal N., Mugale M.N., Mishra P.R. (2024). YIGSR-functionalized hybrid exosomes spatially target dasatinib to laminin receptors for precision therapy in breast cancer. Adv. Healthc. Mater..

[72] Wang X., Wang H., Zhao H., Li N., Li J., Zhang H., Di L. (2024). Targeted delivery of hybrid nanovesicles for enhanced brain penetration to achieve synergistic therapy of glioma. J. Control. Release.

[73] Wen X., Zuo Z., Yang L., Qi X., Wei Z., Xu S., Li J., Luo X., Hu G., Liao Z. (2025). Bortezomib-loaded hybrid liposome inducing pyroptosis for targeted therapy against colorectal cancer. Drug Deliv. Transl. Res..

[74] Li Z., Cai Z., Wang S., Zhu S., Liu C., Wang C. (2025). Construction of biomimetic hybrid nanovesicles based on M1 macrophage-derived exosomes for therapy of cancer. Chin. Chem. Lett..

[75] Duan L., Xu X., Xu Z., Qin X., Zhou X., Xiao Y., Liang Y., Xia J. (2021). Exosome-mediated delivery of gene vectors for gene therapy. Nanoscale.

[76] Zhen X., Li Y., Yuan W., Zhang T., Li M., Huang J., Kong N., Xie X., Wang S., Tao W. (2024). Biointerface-engineered hybrid nanovesicles for targeted reprogramming of tumor microenvironment. Adv. Mater..

[77] Sulthana S., Shrestha D., Aryal S. (2024). Maximizing liposome tumor delivery by hybridizing with tumor-derived extracellular vesicles. Nanoscale.

[78] Abdel-Bar H.M., Tandiono S., Liam-Or R., Cheung C.C.L., Hassuneh O.W.M., Lyu Q., Qin Y., Han S., Rouatbi N., Wang J.T. (2025). Optimizing exosome lipid hybrid nanoparticles for enhanced siRNA delivery and improved therapeutic anticancer efficacy in vivo. ACS Nano.

[79] Ma Y., Zhang J., Rui Y., Rolle J., Xu T., Qian Z., Gu Y., Li S. (2021). Depletion of glioma stem cells by synergistic inhibition of mTOR and c-Myc with a biological camouflaged cascade brain-targeting nanosystem. Biomaterials.

[80] Cheng L., Zhang X., Tang J., Lv Q., Liu J. (2021). Gene-engineered exosomes-thermosensitive liposomes hybrid nanovesicles by blockade of CD47 signal for combined photothermal therapy and cancer immunotherapy. Biomaterials.

[81] Li L., He D., Guo Q., Zhang Z., Ru D., Wang L., Gong K., Liu F., Duan Y., Li H. (2022). Exosome-liposome hybrid nanoparticle codelivery of TP and miR497 overcomes chemoresistant ovarian cancer. J. Nanobiotechnol..

[82] Zhou H., Zhu C., Li Y., Zhao F., Feng Q., Liu S., Jia S., Ji J., Ye L., Zhai G. (2025). Exosome/liposome hybrid nanovesicles for enhanced phototherapy and boosted anti-tumor immunity against melanoma. Eur. J. Med. Chem..

[83] Tong Q., Li K., Huang F., Dai Y., Zhang T., Muaibati M., Abuduyilimu A., Huang X. (2023). Extracellular vesicle hybrid plasmid-loaded lipid nanovesicles for synergistic cancer immunotherapy. Mater. Today Bio.

[84] Zhou X., Tang W., Zhang Y., Deng A., Guo Y., Qian L. (2024). Liposome-exosome hybrids for in situ detection of exosomal miR-1246 in breast cancer. Analyst.

[85] Wu S., Yun J., Tang W., Familiari G., Relucenti M., Wu J., Li X., Chen H., Chen R. (2023). Therapeutic m^6^A eraser ALKBH5 mRNA-loaded exosome-liposome hybrid nanoparticles inhibit progression of colorectal cancer in preclinical tumor models. ACS Nano.

[86] Liu Y., Li M., Gu J., Huang H., Xie H., Yu C., Roy S., Chen X., Kuang T., Zhang Y. (2025). Engineering of exosome-liposome hybrid-based theranostic nanomedicines for NIR-II imaging-guided photothermal therapy of glioblastoma. Colloids Surf. B Biointerfaces.

[87] Barone A., Zimbo A.M., d’Avanzo N., Tolomeo A.M., Ruga S., Cardamone A., Celia C., Scalise M., Torella D., La Deda M. (2023). Thermoresponsive M1 macrophage-derived hybrid nanovesicles for improved in vivo tumor targeting. Drug Deliv. Transl. Res..

[88] Kang K., Zhang Y., Zhou X., Yu Y., Zhu N., Cheng J., Yi Q., Wu Y. (2023). Hybrid extracellular vesicle–liposome camouflaged magnetic vesicles cooperating with bioorthogonal click chemistry for high-efficient melanoma circulating tumor cell enrichment. Adv. Healthc. Mater..

[89] Mizenko R.R., Feaver M., Bozkurt B.T., Lowe N., Nguyen B., Huang K.W., Wang A., Carney R.P. (2024). A critical systematic review of extracellular vesicle clinical trials. J. Extracell. Vesicles..

[90] Lu S., Cui Q., Zheng H., Ma Y., Kang Y., Tang K. (2023). Challenges and Opportunities for Extracellular Vesicles in Clinical Oncology Therapy. Bioengineering.

[91] Chen Y.F., Luh F., Ho Y.S., Yen Y. (2024). Exosomes: A review of biologic function, diagnostic and targeted therapy applications, and clinical trials. J. Biomed. Sci..

